# Effects of Lung Protective Ventilation on the Cognitive Function Level of Patients with Esophageal Cancer

**Published:** 2019-02

**Authors:** Shuming WEI, Shengde LI, He DONG, Wenming XIAO, Mingsheng LI, Haichen CHU

**Affiliations:** 1. Department of Medicine, Qingdao University, Qingdao 266000, P.R. China; 2. Department of Anesthesiology, The Affiliated Hospital of Qingdao University, Qingdao 266000, P.R. China; 3. Department of Oncology, Taian Central Hospital, Taian 271000, P.R. China

**Keywords:** Esophageal cancer, Lung protective ventilation, Cognitive function

## Abstract

**Background:**

We intended to investigate the effects of lung protective ventilation on the cognitive function level of patients with esophageal cancer.

**Methods:**

Overall, 132 patients with esophageal cancer admitted to Taian Central Hospital, Taian China from January 2013 to January 2017 were enrolled in the study. According to the random number table method, they were divided into observation group and control group, 66 cases each. All patients underwent general anesthesia for thoracoscopic esophageal cancer radical operation, and lung protective ventilation and conventional positive pressure ventilation were used respectively. The levels of SOD, NSE and MDA, MMSE score and WMS memory quotient in the blood of the elbow vein were compared between the two groups at different times.

**Results:**

The levels of SOD and MMSE in the observation group at T2 and T3 were higher than those in the control group, and the NSE and MDA levels were lower than those in the control group (*P*=0.013, 0.033, 0.015, 0.044, 0.034, 0.029, 0.014, 0.017). The incidence of postoperative cognitive impairment was lower in the observation group than in the control group (*P*=0.007). The WMS memory quotient scores of the patients in the T2, T3, and T4 observation groups were higher than those in the control group (*p*=0.009, 0.032, 0.040).

**Conclusion:**

Lung protective ventilation can reduce the oxidative stress injury for patients and improve their postoperative cognitive function and memory ability.

## Introduction

Esophageal cancer is a common malignant tumor. In recent years, with the change of dietary habits and structure of Chinese residents, the incidence rate of esophageal cancer has been increased year by year. After the onset of this disease, it will not only invade the esophageal mucosa tissue, but also lead to tumor metastasis, which will exert serious impacts on the living quality and life of patients (1). The clinical treatments for esophageal cancer include radical resection of esophageal cancer, drug chemotherapy and radiation therapy, among which the radical resection of esophageal cancer is the most effective treatment clinically (2).

Cognitive impairment is a common complication of patients after surgery which is a mild neurocognitive disorder mainly manifested as decreased cognitive functions in the aspects of attention, memory and learning ability of patients. Its mechanism is not clear yet, but it is considered that the barotrauma made by the inappropriate parameters of mechanical ventilation during operation that can cause the excessive expansion or collapse of pulmonary alveoli. And then the body oxidative stress is induced and develops into excessive reaction, which brings oxidative stress injury. As a result, the cognitive function of patients would be affected. Based on the low volume of tidal air ventilation and combined with lung ventilation scheme, the lung protective ventilation can attenuate the lung injury and reduce systemic oxidative stress (3–5).

Therefore, the effects of lung protective ventilation on the cognitive function of patients with esophageal cancer were explored in this study in order to provide references for later treatment. Now it is analyzed and reported as follows:

## Materials and Methods

### General data

Overall, 132 patients with esophageal cancer admitted to Taian Central Hospital, Taian China from January 2013 to January 2017 were selected as subjects. There were 66 patients in observation group, including 37 males and 39 females. They were aged 18 to 63 yr old with an average age of (48.28±4.82) yr old. The body mass index was (20.53±0.53) kg/m2; there were 66 patients in control group, including 35 males and 31 females. They were aged 18 to 62 yr old with an average age of (47.33±4.91) yr old. The body mass index was (20.81±0.72) kg/m2. The differences in general data of patients between the two groups were not statistically significant (*P*>0.05), so the data were comparable.

All subjects and their families agreed to participate in the experiment and signed an informed consent form. This experiment has been approved by the Ethics Committee of Taian Central Hospital.

Inclusion criteria: 1) Patients were diagnosed with esophageal cancer clinically; 2) patients were successfully treated with thoracoscopic esophageal cancer radical surgery under general anesthesia in our hospital; 3) patients aged ≥ 18 yr old; 4) Patients volunteered to participate in this study and signed the informed consent.

Exclusion criteria: 1) patients complicated with other severe acute or chronic diseases; 2) patients complicated with other diseases which can cause cognitive impairment; 3) patients with histories of administration of narcotic analgesics and antipsychotic drugs; 4) patients with severe mental illness.

### Methods

Patients in the two groups were both treated with thoracoscopic esophageal cancer radical surgery under general anesthesia. Patients in control group were treated with conventional positive pressure ventilation (frequency: 14 times/min; tidal volume: 8 mL/kg; percentage of inhaled oxygen concentration: 80%). Patients in observation group were treated with lung protective ventilation [frequency: 16 times/min; tidal volume: 6 mL/kg; percentage of inhaled oxygen concentration: 80%; positive end-expiratory pressure (PEEP): 8cmH2O], combined with manual lung recruitment every half an hour at the same time (the plateau pressure is kept at 30 cmH2O, time: 30 s).

### Observation indexes

The levels of superoxide dismutase (SOD), neuron-specific enolase (NSE) and malondialdehyde (MDA), mini mental state examination (MMSE) score and memory quotient score of Wechsler Memory Scale (WMS) in the elbow venous blood of two groups of patients at T1, T2, T3 and T4 were compared. 1) 5 mL of elbow venous blood of patients were taken and centrifuged at the rate of 3000 r/min. Clear supernatants were removed. SOD activity was detected through xanthine oxidase method. Concentration of MDA was determined through thiobarbituric acid method. Enzyme linked immunosorbent assay (ELISA) was used to determine the concentration of NSE. 2) Mini mental state examination (MMSE) score and memory quotient score of Wechsler Memory Scale (WMS): The patients were assessed by non-operating physicians at different moments, and the evaluation time was fixed at 4 p.m. every day. It was considered to be cognitive impairment if the MMSE score was less than 27. The WMS memory quotient test was performed at the same time, including instantaneous memory, short-term memory and long-term memory. The memory quotient was calculated according to the scores. The higher the score of memory quotient was, the better memory of patients would be.

### Statistical methods

The statistical analysis was conducted with Statistical Product and Service Solutions (SPSS) 20.0 software. The *t* test was used for measurement data and *x2* test was applied for count data. The difference would be statistically significant if *P*<0.05.

## Results

### Levels of SOD, NSE and MDA at different moments

Differences in levels of SOD, NSE and MDA at T1 and T4 between two groups of patients were not statistically significant (*P*=0.077, 0.096, 0.056, 0.067, 0.171, 0.088). Compared with those at T1, levels of SOD at T2 and T3 in two groups of patients were decreased, and levels of NSE and MDA were increased. Level of SOD in observation group was higher than that in control group. Levels of NSE and MDA in the former were lower than those in the latter. The differences were statistically significant (*P*=0.013, 0.033, 0.034, 0.029, 0.014, 0.017) (Table 1).

**Table 1:** Levels of SOD, NSE and MDA at different moments

***Group***	***SOD (U/mL)***				***NSE (μg/L)***				***MDA (mmol/mL)***			
	***T1***	***T2***	***T3***	***T4***	***T1***	***T2***	***T3***	***T4***	***T1***	***T2***	***T3***	***T4***
Observation group (n=66)	431.33±43.28	412.55±36.41	406.38±29.16	429.74±27.43	9.30±1.49	12.59±2.96	15.30±2.38	10.13±3.58	4.89±0.13	6.16±0.25	5.10±0.26	5.07±0.35
Control group (n=66)	428.47±32.11	398.45±27.48	351.46±41.33	435.55±35.28	9.12±2.66	19.58±3.68	21.57±3.47	9.69±2.87	5.02±0.24	6.69±0.35	5.93±0.44	4.99±0.28
*t* value	1.813	2.561	2.377	1.663	1.947	2.352	2.435	1.915	1.619	2.559	2.537	1.689
*P* value	0.077	0.013	0.033	0.096	0.056	0.034	0.029	0.067	0.171	0.014	0.017	0.088

### Comparisons of MMSE scores at different moments

Differences in MMSE scores at T1 and T4 between two groups of patients were not statistically significant (*P*=0.137, 0.061). Compared with those at T1, MMSE scores at T2 and T3 in two groups of patients were decreased. The MMSE scores of patients in observation group were higher than those in control group. The differences were statistically significant (*P*=0.015, 0.044). The incidence rates of cognitive impairment for patients after operation in observation group and control group were 12.12% (8/66) and 22.27% (15/66), respectively. The incidence rate in control group was higher than that in observation group. The difference were statistically significant (*P*=0.007) (Table 2).

**Table 2:** Comparison of MMSE scores at different moments (scores)

***Group***	***T1***	***T2***	***T3***	***T4***
Observation group (n=66)	28.94±1.11	27.81±0.67	28.71±0.68	29.10±0.98
Control group (n=66)	29.04±1.20	25.90±0.78	27.02±0.71	28.89±0.73
*t* value	1.629	2.557	2.037	1.931
*P* value	0.0137	0.015	0.044	0.061

### Comparisons of WMS memory quotient scores at different moments

Differences in WMS memory quotient scores at T1 between two groups of patients were not statistically significant (*P*=0.099). Compared with those at T1, the WMS memory quotient scores at T2, T3 and T4 were decreased in two groups of patients. The WMS memory quotient scores in observation group were higher those in control group. The differences were statistically significant (*P=*0.009, 0.032, 0.040) (Table 3).

**Table 3:** Comparison of WMS memory quotient scores at different moments (scores)

***Group***	***T1***	***T2***	***T3***	***T4***
Observation group (n=66)	90.94±3.23	75.47±2.78	78.22±3.19	81.33±3.54
Control group (n=66)	90.22±3.47	71.67±3.81	75.62±3.04	78.65±3.42
*t* value	1.649	2.678	2.389	2.147
*P* value	0.099	0.009	0.032	0.040

## Discussion

The acute pain of patients with esophageal cancer after surgical treatment will affect the recovery of various organs and systematic functions of patients, leading to poor postoperative mental state and respiratory function (6). Surgical trauma will cause the body stress response. Stress trauma will activate the hypothalamic-pituitary-adrenal cortical axis function. The disorder of neurotransmitter systems such as center 5-hydroxytryptamine (5-HT), noradrenaline (NE) and acetylcholine (Ach) of patients after surgery will occur and result in increased glucocorticoid concentration during the circulation. The release volume of inflammatory cytokines in the body such as inter-leukin-1 (IL-1), interleukin-2 (IL-2), interleukin-6 (IL-6) and tumor necrosis factor (TNF) are also increased (7). Therefore, the inflammatory response of the central nervous system of patients may affect the hippocampal function so as to cause cognitive dysfunction or mental disorder of patients. Studies have pointed out that using 4 mL/kg-6 mL/kg tidal air can effectively protect lung function during one-lung ventilation (8). Therefore, in this study, patients in the observation group were given 6 mL/kg tidal air, combined with lung recruitment, in order to improve their levels of postoperative cognitive function.

SOD is an endogenous free radical scavenger. The production of free radicals decreases SOD content. SOD level in serum can indirectly reflect the conditions of free radicals (9); MDA is produced by the decomposition of lipid peroxide, whose level in serum can indirectly reflect the degree of body lipid peroxidation reaction in patients (10). Therefore, the impairment degree of cells made by oxygen-derived free radicals can be indirectly reflected through the detection of MDA level and SOD activity in patients. NSE is an acid protease specific to neuroendocrine cells and neurons. NSE content in the human body under normal circumstances is very low. But if the neurons suffered stimulations such as ischemia or hypoxia, the integrity of their cell membranes would be damaged. At this time, the NSE inside the cell membrane will be released out, and it will pass through blood brain barrier and enter into serum.

The results of this study showed that the differences in levels of SOD, NSE and MDA at T1 and T4 between two groups of patients were not statistically significant. Compared with those at T1, SOD levels at T2 and T3 in two groups of patients were decreased; levels of NSE and MDA were increased. SOD level in observation group was higher than that in control group; the levels of NSE and MDA in the former were lower than those in control group. The differences were statistically significant. This indicates that the lung protective ventilation taken for patients in the observation group can effectively reduce body oxidative stress injury, thereby improving the level of cognitive function.

MMSE score is one of the commonly used methods for effective evaluation of cognitive function. After the exclusion of the interferences of abnormal consciousness and emotion, the cognition of brain function becomes the focus. The results of this study indicated that compared with those at T1, MMSE scores at T2 and T3 were decreased in two groups of patients. The MMSE scores in observation group were higher than those in control group. The differences were statistically significant. The incidence rates of postoperative cognitive impairment of patients in the observation group and control group were 12.12% (8/66) and 22.72% (15/66), respectively. The control group had a higher incidence rate than that of the observation group, and the difference was statistically significant (*P*=0.007). This suggested that the cognitive function level of patients is greatly improved after they were treated with lung protective ventilation. The reason for that is 6 mL/kg tidal air can reduce barotrauma, improve the lung dynamic and static compliances, maintain oxygen saturation concentration, and increase the ventilation for dead space and control PEEP properly so as to effectively prevent the pulmonary atelectasis and alveolar collapse and to keep the end-expiratory pulmonary alveoli open. Finally, the collapsed pulmonary alveoli is driven to be open again (11, 12).

Therefore, compared with patients treated with 8 mL/kg tidal air in control group, patients in observation group have better performances in the aspects of alveolar recruitment, pulmonary oxygen saturation and lung functions after operation.

## Conclusion

Lung protective ventilation can reduce oxidative stress injury of patients and improve their postoperative cognitive function and memory ability. It can be further popularized and used in clinical practice.

## Ethical considerations

Ethical issues (Including plagiarism, informed consent, misconduct, data fabrication and/or falsification, double publication and/or submission, redundancy, etc.) have been completely observed by the authors.
